# Relationship between school support and digital teaching adaptation among physical education teachers: the mediating roles of self-efficacy, digital teaching beliefs and teaching intention

**DOI:** 10.3389/fpsyg.2026.1786873

**Published:** 2026-03-18

**Authors:** Jing Wang, Yuanwei Li, Mingyu Wang, Junxian Zhang, Jasmin Hutchinson, Defang Sun, Wenjing Zeng

**Affiliations:** 1 School of Physical Education, Hangzhou Normal University, Hangzhou, Zhejiang, China; 2 Department of Physical Education and Health Education, Springfield College, Springfield, MA, United States; 3 Jing Hengyi School of Education, Hangzhou Normal University, Hangzhou, Zhejiang, China; 4 School of International Studies, Hangzhou Normal University, Hangzhou, Zhejiang, China; 5 Department of Exercise Science, School of Physical Education, Springfield College, Springfield, MA, United States; 6 Chinese Education Modernization Research Institute of Hangzhou Normal University (Zhejiang Provincial Key Think Tank), Hangzhou, Zhejiang, China

**Keywords:** digital teaching beliefs, digital teaching intention, digital teaching adaptation, physical education teachers, school support, self-efficacy, social cognitive theory

## Abstract

**Introduction:**

With rapid advances in technology, school based physical education is undergoing a systemic reconstruction in its organizational structure and educational model, with growing focus on teachers' adaptability to dynamic digital teaching environments. The digital adaptation of physical education teachers remains insufficiently theorized, particularly given the contextualized, demonstration-based and interaction-intensive nature of PE instruction, highlighting the need for systematic investigation into its key determinants and developmental pathways. Grounded in the Social Cognitive Theory, this study assessed self-efficacy, digital teaching beliefs, and digital teaching intention to examine how school support is associated with teachers' digital teaching adaptation.

**Methods:**

A total of 409 physical education teachers from multiple provinces in China participated in this study. Data were collected using standardized questionnaires measuring school support, self-efficacy, digital teaching beliefs, digital teaching intention, and digital teaching adaptation. The data were analyzed through correlation analysis and structural equation modeling.

**Results:**

The results indicate that, first, school support significantly and positively predicts digital teaching adaptation among PE teachers. Second, self-efficacy partially mediates in the relationship between school support and teacher's digital teaching adaptation. Third, a chain mediation effect exists whereby self-efficacy and digital teaching intention sequentially mediate the relationship between school support and digital teaching adaptation. Similarly, digital teaching beliefs and digital teaching intention also form a chain mediation pathway. Furthermore, self-efficacy, digital teaching beliefs, and digital teaching intention jointly constitute a serial mediation mechanism linking school support to digital teaching adaptation.

**Discussion:**

These findings clarify how school support is associated with teachers' digital teaching adaptation through key psychological factors in the context of educational digital transformation. The study provides empirical evidence and practical insights for the advancement of digital education reform.

## 1 Introduction

Driven by the ongoing digitalization of education, the organizational structure, learning environment, and pedagogical model of school-based Physical Education (PE) are undergoing systemic and ecological transformation (Calderón and Meroño, 2020). As this transformation unfolds, a range of digital technologies to enhance pedagogy in physical education (AL-Sinani and Al Taher, 2023; Martín-Rodríguez and Madrigal-Cerezo, 2025), such as intelligent equipment-assisted instruction (Cui et al., 2025), blended virtual-physical activity scenarios (Li et al., 2025), digital performance assessment systems (He et al., 2024), and AI-based risk detection in movement (Chen and Dai, 2024; Kim et al., 2025), are becoming increasingly embedded in everyday PE practices. This shift requires teachers to continuously refine instructional strategies, effectively utilize diverse digital tools, and creatively integrate digital technologies throughout the teaching process (Gülünay and Coşar, 2025). As a result, PE teachers' digital teaching adaptation not only serves as a crucial foundation for fostering students' digital physical literacy, but also represents a vital driving force in the modernization of school physical education.

PE teachers face several significant challenges in adapting to digital teaching. These include limited skills in health data analysis (Greve et al., 2022), notable deficiencies in both digital teaching beliefs and competence (Saiz-González et al., 2025), and psychological resistance to digitalization (Wohlfart et al., 2024). Such obstacles hinder the pace and effectiveness of teachers' digital transformation, underscoring the urgent need to support their adaptation to digital teaching through technology integration (AlKasasbeh and Amawi, 2024). Therefore, exploring how to support and promote physical education teachers‘ digital teaching adaptation has become a dual priority in both academic research and practical application.

Previous research suggests that the digital adaptation process is influenced by a complex interplay of factors. At the individual level, technical capabilities, behavioral patterns, and psychological states all play a role (Østerlie et al., 2025). Beyond these individual factors, external factors, particularly school support, further shape teachers' ability to adapt to digital teaching environments (Liu et al., 2025). As the primary organizational context for teacher development, schools play a vital role in supporting teachers through institutional policies, resource allocation, training opportunities and cultural environment, all of which form essential conditions for teachers' technology adoption and instructional transformation (Bhagat et al., 2025). Research has consistently shown that school support plays a vital role in teachers' adoption of technology (Scherer et al., 2019) and is a key external factor shaping how technology is integrated into their teaching (Inan and Lowther, 2010).

Although teachers' digital adaptation process appears to involve several factors, previous research has focused on analyzing the effect of a single factor, neglecting the complex and comprehensive effects between school support and individual factors of teachers. There is also a lack of clear theoretical and empirical explanations on how school support is associated with PE teachers' digital teaching adaptation. Social Cognitive Theory (SCT), proposed by Bandura (1977, 1986) asserts that individual behavior is influenced in a reciprocal manner by both environmental and individual factors. This framework provides an effective perspective for analyzing how school support is associated with PE teachers' digital teaching adaptation based upon interacting factors such as the school environment and teachers' individual cognition and behavior in educational contexts.

This study uses SCT to explore how school support is associated with PE teachers' digital teaching adaptation. Specifically, three core cognitive variables, derived from SCT and repeatedly validated in the field of teacher technology adaptation, are examined as potential mediators (Shi et al., 2025; Alberola-Mulet et al., 2021; Müller and Leyer, 2023). The first variable, self-efficacy, refers to confidence in one's ability to perform a specific behavior. In this case, the confidence of teachers when facing digital teaching challenges, may serve as a crucial cognitive bridge linking external environment to individual motivation. The second, teaching belief, which refers to the teachers' value judgment toward regarding digital teaching's potential to enhance their teaching effectiveness, is a comprehensive reflection of individual motivation. The third, teaching intention, which is the teachers' intent to participate in digital teaching practice and serves as the key condition for individual cognitive factors to be transformed into behavior. These individual factors work together with school support as a key environmental factor to influence the behavior of teachers, such as technology integration and digital teaching adaptation, forming a complete closed loop of the “environment-individual-behavior” interaction mechanism proposed in SCT.

In summary, this study posits that teacher digital teaching adaptation is a dynamic behavior co-constructed through external support systems and internal cognitive beliefs. We propose an integrative framework to explain this process, involving multiple key pathways: a direct association of school support and several indirect associations through teachers' self-efficacy and value recognition. Based on this, the core research question of this study is as follows:

How does school support interact with PE teachers' self-efficacy, digital teaching beliefs and intention in relation to their digital teaching adaptation?

## 2 Literature review and model construction

### 2.1 Teachers' digital teaching adaptation

Teaching adaptation refers to the deliberate adjustments that teachers make in instructional strategies, content delivery, and assessment methods in response to environmental or technological changes (Skulmowski and Rey, 2020). As digital education becomes increasingly prevalent, teachers must not only acquire the technical competence to effectively operate intelligent technologies, but also achieve emotional and pedagogical integration with these tools (Zhao et al., 2024; Zhao, 2016). For PE teachers, within the digital environment, they need to combine the characteristics of PE discipline with specific teaching contexts, effectively integrate and apply digital technology to construct teaching scenarios, design content, and develop and implement personalized exercise plans tailored to students' physical fitness differences. Therefore, PE teachers' digital teaching adaptation in this study refers to the comprehensive process of teachers adapting to this new teaching environment and teaching reform.

### 2.2 School support: the critical external force for promoting teachers' digital teaching adaptation

School support has always been a critical environmental factor in the professional development of PE teachers, which encompasses resources, institutional arrangements, professional learning, and a supportive culture. Under new technological conditions, school support has become an essential driver of educational reform through the transformation and upgrading of school organizational culture, educational objectives, and administrative mechanisms (Tian and Zhang, 2020). In the context of intelligent and precise instructional research, school support is conceptualized as a multi-dimensional service mechanism involving technology infrastructure, administrative assistance, professional development opportunities, and collaborative frameworks, all of which are vital in achieving educational goals (Zheng et al., 2023). Drawing on the Total Quality Management (TQM) theory, school support in this study is defined as the system support that PE teachers received in digital teaching environments, which includes personnel, infrastructure, curriculum, institutional and environmental support.

Systemic school support in terms of technology, training, and cultural environment has been shown to significantly enhance teachers' effective integration and adaptation to digital teaching (Lattke et al., 2025). Empirical studies indicate that school-based training support plays a crucial role in promoting teachers' acceptance of digital technologies and supporting their technical adaptation (Wohlfart and Wagner, 2023). Moreover, the school culture that promotes digital instruction not only motivates teachers to adopt digital tools in their teaching but also supports the development of their digital teaching identity, thereby enhancing their overall adaptability (Collie et al., 2020). Based on these, the following hypothesis was proposed.

H1: School support is significantly and positively associated with PE teachers' digital teaching adaptation.

### 2.3 Self-efficacy, digital teaching beliefs and digital teaching intention

Self-efficacy refers to an individual's belief or confidence in their ability to successfully perform a specific behavior to achieve a desired outcome (Bandura, 1977). In educational practice, especially in the context of digital teaching, teachers often need to cope with various challenges and difficulties. Their confidence in addressing these challenges and in effectively using digital tools and technologies to support teaching, referred to as teachers' digital self-efficacy, is therefore of critical importance (Barkati et al., 2024; Troesch and Bauer, 2017; Zheng and Xiao, 2024). In this study, PE teachers' self-efficacy refers to their perceived confidence in conducting digital teaching in digital teaching contexts, specifically the confidence in their ability to effectively use digital tools to facilitate teaching, enhance student engagement, and achieve intended learning outcomes when facing pedagogical and technological challenges.

Teachers' confidence in teaching is not solely driven by intrinsic motivation, but also deeply rooted in the structural resources and interpersonal support provided by schools (Xafakos and Malafantis, 2025). Greater access to digital instructional tools enhances teachers' self-efficacy in digital teaching (Fan et al., 2023), which in turn strengthens their capacity to integrate technology effectively into instruction (Talosa et al., 2025). Furthermore, the improvement of self-efficacy related to digital literacy has also been linked to increased work engagement and greater psychological adaptability in digital teaching environments (Fan et al., 2023). Based on these, the following hypotheses were proposed.

H2: School support is significantly and positively associated with PE teachers' self-efficacy.
H3: Self-efficacy is significantly and positively associated with PE teachers' digital teaching adaptation.

Teachers' beliefs are individual interpretations and experience-based propositions that guide how they perceive and address problems in teaching and learning (Ferguson and Bråten, 2022). In the context of digital teaching, the fundamental cognitive and judgment system held by teachers regarding the value, utility, and practical principles of digital teaching constitutes their digital teaching beliefs (Sarra et al., 2025). In this study, teachers' digital teaching beliefs refer to the comprehensive judgment of PE teachers on the value of carrying out digital teaching in physical education.

Research has shown that schools can significantly enhance teachers' positive beliefs about digital teaching by offering model lessons, structured training and fostering a supportive school culture (Aumann et al., 2025). Meanwhile, self-efficacy has been identified as an important factor influencing teachers' instructional beliefs, which can significantly strengthens teachers' teaching beliefs (König et al., 2020; Lee et al., 2013). And teaching beliefs play a critical role in determining their ability to adapt to technology-integrated teaching environments (Tondeur et al., 2017). Based on these findings, the following hypotheses are proposed.

H4: School support is significantly and positively associated with PE teachers' digital teaching beliefs.
H5: Self-efficacy is significantly and positively associated with PE teachers' digital teaching beliefs.
H6: Digital teaching beliefs are significantly and positively associated with PE teachers' digital teaching adaptation.

Furthermore, Teaching intention is a psychological state that reflects a teacher's desire to learn, experiment, and try new practices, and serves as a prerequisite for actual teaching behavior to occur (Van Eekelen et al., 2006). Teachers' intention to use digital tools refers to their motivational willingness to integrate technology into their instructional practices (Joo et al., 2018). In this study, digital teaching intention is defined as the psychological tendency and behavioral inclination of PE teachers to proactively learn, actively apply, and creatively integrate digital tools and technologies into their teaching practices.

When schools provide adequate technical resources and necessary training, teachers' intention to engage in digital teaching can be significantly improved (Saiz-González et al., 2025). In PE, teachers' instructional intentions are shaped by a combination of factors, including curriculum beliefs, self-efficacy, and subjective norms (Brown, 2023). Beliefs and attitudes toward specific content areas also play a critical role, significantly influencing teachers' intention to teach such topics (Lai et al., 2018). Furthermore, teachers' willingness to change has been identified as a key predictor of professional adaptability, supporting their capacity to adjust to evolving teaching demands (Herawati et al., 2022). Based on these findings, the following hypotheses are proposed.

H7: School support is significantly and positively associated with PE teachers' digital teaching intention.
H8: Self-efficacy is significantly and positively associated with PE teachers' digital teaching intention.
H9: Digital teaching beliefs are significantly and positively associated with PE teachers' digital teaching intention.
H10: Digital teaching intention is significantly and positively associated with PE teachers' digital teaching adaptation.

**Figure 1:**
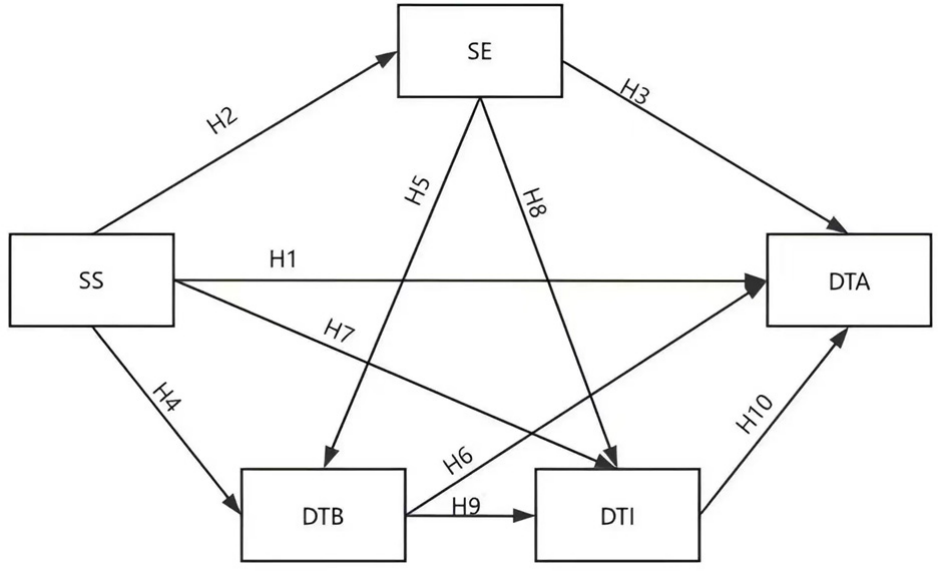
Theoretical Hypothesis Model. SS school support, SE self-efficacy, DTB digital teaching beliefs, DTI digital teaching intention, DTA digital teaching adaptation.

Based on above hypotheses, this study proposes a theoretical model of serial mediation between school support and digital teaching adaptation (Figure 1).

## 3 Methods

### 3.1 Participants

A stratified random sampling method was used to administer a questionnaire survey to PE teachers across different educational levels and regions in eastern, central, and western China. The survey was conducted online using the Wenjuanxing platform (https://www.wjx.cn), through which a total of 409 valid responses were collected. The demographic details as follows: In terms of gender, male respondents were predominant, constituting 60.60% (*n* = 248) of the total sample, which was slightly over the number of females (*n* = 161; 39.40%). In terms of age distribution, the most represented group was teachers aged 30 or younger (*n* = 155; 37.90%), followed by aged 31 to 40 (*n* = 119; 29.10%) and then aged 41 to 50 (*n* = 94; 23.00%). In contrast, only a small proportion of respondents were over 50 years old (*n* = 41; 10.00%). Regarding educational background, postgraduate degrees were the most common qualification (*n* = 224; 54.77%), followed by bachelor's degree or below (*n* = 185; 45.23%). In terms of teaching level, 51.30% of the teachers were from the basic education stage, while 48.70% were from the higher education stage.

### 3.2 Measurement instruments

#### 3.2.1 School support scale

The school support scale used in this study was designed based on the Total Quality Management (TQM) theory to capture multiple dimensions of school support, including personnel support, infrastructure support, curriculum support, institutional arrangements, and environmental support. Specific scale items was adapted from the School Support Scale (Zhao et al., 2022) and the Teacher Social Support Scale (Qi and Zhao, 2024). The scale was adapted to align with the specific context of PE teachers. The initial scale includes items such as “School leaders and colleagues around me possess strong information technology based teaching competence” “The intelligent hardware and software facilities for physical education in my school meet my teaching needs” “The digital resources or technologies introduced by my school meet my physical education teaching needs” “My school has established a relatively comprehensive digital physical education teaching management system” “My school emphasizes the construction of a high-quality digital teaching environment for physical education.” All items were measured using a five-point Likert scale (1 = strongly agree, 5 = strongly disagree). Through confirmatory factor analysis (CFA) and structural equation modeling (SEM), it was found that the individual model fitting indicators were not satisfactory. A detailed analysis reveals that the residuals of individual items were not independent, which indicates that the viewpoints expressed in these items were relatively similar. Based on the principles of model simplification and residual independence, and guided by modification indices (MI > 10, *p* < 0.001), theoretical considerations and the statement of items, five items with high MI and similar viewpoints to other items were removed in sequence (Detailed item removal procedures are provided in Supplementary Tables A1, A2, B2). The final scale demonstrated good internal consistency, with a Cronbach's α of 0.943 and a KMO value of 0.909, indicating high reliability and validity.

#### 3.2.2 Digital self-efficacy scale

This scale was adapted from the Digital Self-Efficacy Scale (Kim et al., 2025) to suit the specific context of PE teachers. The scale, comprises three items, including: “I believe I can easily learn how to conduct digital physical education teaching” “I am confident in my ability to effectively carry out digital physical education teaching” and “I can independently solve the difficulties encountered in digital physical education teaching.” All items were measured using a five-point Likert scale (1 = strongly agree, 5 = strongly disagree). The scale demonstrated high reliability and validity, with a Cronbach's α of 0.930 and a KMO value of 0.751, indicating good internal consistency and construct validity.

#### 3.2.3 Digital teaching beliefs scale

This scale was adapted from the Digital Teaching Beliefs Scale (Zhang, 2023), based on the specific context of PE teachers. The scale included two dimensions: digital teaching identification and digital teaching values. It originally consisted of six items, including: “I believe that a digital teaching environment can help reduce the work pressure of physical education teachers” “I believe that digital physical education teaching can promote students' comprehensive development through digital technologies and implement the fundamental task of cultivating moral character” etc. All items were rated using a five-point Likert scale (1 = strongly agree, 5 = strongly disagree). The CFA revealed that the individual model fitting indicators were not satisfactory due to the non-independence of the residuals of individual items. To simplify the model and adhere to the principle of residual independence, two items that have an inclusive relationship or similar viewpoints with other items were sequentially removed based on the results of modification indices and the statement of items (Detailed item removal procedures are provided in Supplementary Tables A3, A4). The final version of the scale demonstrated strong reliability and validity, with a Cronbach's α of 0.937 and a KMO value of 0.867.

#### 3.2.4 Digital teaching intention scale

A self-developed scale was constructed in this study to measure digital teaching intention. The scale consists of three items, including: “I will actively learn digital technology resources related to education and teaching” “I will actively use digital technology resources in physical education teaching practice” “I will actively integrate digital technology resources into physical education teaching to promote instructional innovation.” All items were assessed using a five-point Likert scale (1 = strongly agree, 5 = strongly disagree). The scale demonstrated high reliability and validity, with a Cronbach's α of 0.950 and a KMO value of 0.766.

#### 3.2.5 Digital teaching adaptation scale

This scale was developed by referring to the Social Adaptation Scale for “digital immigrants” (Wang and Li, 2023) and the Digital Literacy Standards for Teachers issued by The Ministry of Education of the People's Republic of China, and by integrating the specific context of PE teachers. The scale originally consisted of three dimensions: technological adaptation, behavioral adaptation and cultural adaptation, and included eight items. Example items included: “I understand the concepts and principles of digital technologies such as the Internet and big data,” “I am able to use digital technology resources to collect, analyze, and assess students' physical education learning data,” and “I can comply with laws, regulations, and ethical norms related to digital activities in physical education teaching.” All items were measured using a five-point Likert scale (1 = strongly agree, 5 = strongly disagree). The CFA and SEM revealed that the model fit well but not excellent due to the non-independence of the residuals of individual items. Based on the principles of model simplification and residual independence, and guided by modification indices (MI > 10, *p* < 0.001), theoretical considerations and the statement of items, three items with high MI and similar viewpoints to other items were removed in sequence (Detailed item removal procedures are provided in Supplementary Tables A5, A6, B2). The final scale demonstrated high reliability and validity, with a Cronbach's α of 0.954 and a KMO value of 0.908.

### 3.3 Data analysis

Firstly, we used SPSS 26.0 to calculate the descriptive statistics and correlations. Secondly, we performed Harman's single-factor test, CFA single-factor and multi-factor comparison and Unmeasured Latent Method Construct (ULMC) to evaluate common method variance (CMV) (Podsakoff et al., 2003). According to previous research (Chen, 2007), changes in fit indices within the ranges (ΔCFI ≤ 0.01, ΔRMSEA ≤ 0.015, andΔSRMR ≤ 0.03) suggest that CMV does not pose a significant threat to the validity of the results. Thirdly, CFA were specified to test the proposed measurement structure underlying the data in Amos 24.0. In addition, we examine the structural model and hypothetical paths in the current study. Several fitting indices were employed to assess the overall model fit. Previous researchers (Hu and Bentler, 1999; MacCallum and Hong, 1997) noted that χ^2^/*df* (< 3), GFI (≥0.90), TLI (≥0.95), CFI (≥0.95), RMSEA (< 0.06), and SRMR (< 0.08) reflect a good fit. Fourth, bootstrap methods with robust standard errors were used to test the significance of mediating effects. The bootstrap approach provided 95% deviation corrected confidence intervals (CIs) for these effects using a resample of 2,000 data. The significance of the indirect effects was indicated if there were no zeros in the CIs. All statistical tests were two-tailed.

## 4 Result

### 4.1 Descriptive statistics and correlation analysis

The results of the descriptive statistics show that the mean scores for all variables ranged from 1.8 to 2.3 indicating that the overall data level in this study was above the average since all items were reverse-coded on a 5-point Likert scale. The results of the correlation analysis show that all variables were significantly and positively correlated with each other, with Pearson correlation coefficients (*r*) ranged from 0.644 to 0.846.

### 4.2 Common method variance

Firstly, in order to eliminate the impact of common method variance, this study adopted anonymous questionnaire surveys, and the questions in questionnaire were concise and easy to understand, without involving questions that may confuse, lead to different interpretations, or be difficult for respondents to answer. Additionally, in the questionnaire design, the header for each dimension had been removed to avoid respondents being influenced by the titles, thereby. Subsequently, this study utilized the Harman's single-factor test, CFA single-factor and multi-factor comparison and ULMC to assess potential common method variance. The results of Harman's single-factor test showed that the model fit indices for the single-factor solution were below acceptable thresholds (χ^2^ = 2203.686, *df* = 170, CFI = 0.795, TLI = 0.771, RMSEA = 0.171, SRMR = 0.069), indicating no substantial common method variance. Five-factor confirmatory factor analysis showed that the model fits well (χ^2^ = 389.107, *df* = 160, CFI = 0.977, TLI = 0.973, RMSEA = 0.059, SRMR = 0.027). Comparing the single-factor model and five-factor model, the fitting of the single-factor model is significantly worse than that of the theoretical five-factor model (Δχ^2^ = 1814.579,Δ*df* = 10, *p* < 0.000), indicating that there was minimal impact of common method variance. Furthermore, the results of ULMC test showed that after adding the common method factor to the original trait factor model, the model fit indices did not significantly improve (χ^2^ = 264.707, *df* = 140, CFI = 0.987, TLI = 0.983, RMSEA = 0.047, SRMR = 0.017). The changes in fit indices is within the specified ranges (ΔCFI = 0.01,ΔTLI = 0.01,ΔRMSEA = 0.012,ΔSRMR = 0.01), indicating that this model was not affected by CMV and there was no significant common method variance.

### 4.3 Confirmatory factor analysis

CFA was performed on the latent constructs of school support, digital self-efficacy, digital teaching intention, digital teaching beliefs, and PE teachers' digital teaching adaptation. Tables 1, 2 show that all standardized factor loadings exceeded the threshold of 0.60 and were statistically significant at *p* < 0.001, with squared multiple correlations (SMC) above 0.36, indicating satisfactory indicator reliability. The composite reliability (CR) for all five constructs ranged from 0.93 to 0.95, well above the commonly accepted reliability threshold of 0.70, confirming high internal consistency and construct reliability. The average variance extracted (AVE) values for all constructs exceeded 0.50, thus reaching the standard threshold and indicating the reliability of the measurement model as well as its good convergent validity (Fornell and Larcker, 1981). Furthermore, the square roots of the AVE values for each latent construct were greater than the corresponding inter-construct standardized correlation coefficients, certifying the robust discriminant validity of the measurement model.

**Table 1:** Composite reliability and convergent validity.

**Construct**	**Item**	**Factor loadings**	**SMC**	**CR**	**AVE**
SS	SS1	0.783	0.613	0.944	0.772
	SS5	0.895	0.770		
	SS6	0.877	0.821		
	SS7	0.906	0.858		
	SS10	0.926	0.800		
SE	SE1	0.908	0.824	0.934	0.824
	SE2	0.935	0.874		
	SE3	0.880	0.774		
DTI	DTI1	0.934	0.873	0.952	0.868
	DTI2	0.946	0.894		
	DTI3	0.914	0.835		
DTB	DTB1	0.900	0.810	0.937	0.789
	DTB3	0.917	0.841		
	DTB4	0.848	0.718		
	DTB5	0.887	0.788		
DTA	DTA1	0.891	0.793	0.955	0.810
	DTA2	0.940	0.884		
	DTA3	0.949	0.901		
	DTA5	0.893	0.797		
	DTA8	0.821	0.673		

**Table 2:** Convergent validity and discriminant validity.

**Construct**	**AVE**	**SS**	**SE**	**DTB**	**DTI**	**DTA**
SS	0.772	(0.879)				
SE	0.824	0.847	(0.908)			
DTB	0.868	0.665	0.701	(0.932)		
DTI	0.789	0.748	0.828	0.807	(0.888)	
DTA	0.810	0.847	0.884	0.754	0.856	(0.900)

Values in parentheses represent the square roots of the AVE.

### 4.4 Structural model and path analysis

This study adopted both absolute fit indices (AGFI, RMSEA, SRMR) and relative fit indices (CFI, TLI) to assess the model fit. The results revealedχ^2^/*df* = 2.432, CFI = 0.977, GFI = 0.913, RMSEA = 0.059, SRMR = 0.026, TLI = 0.973. All indices indicated a good or above-good level of model fit, demonstrating that the structural model used in this study fits the data well.

**Figure 2:**
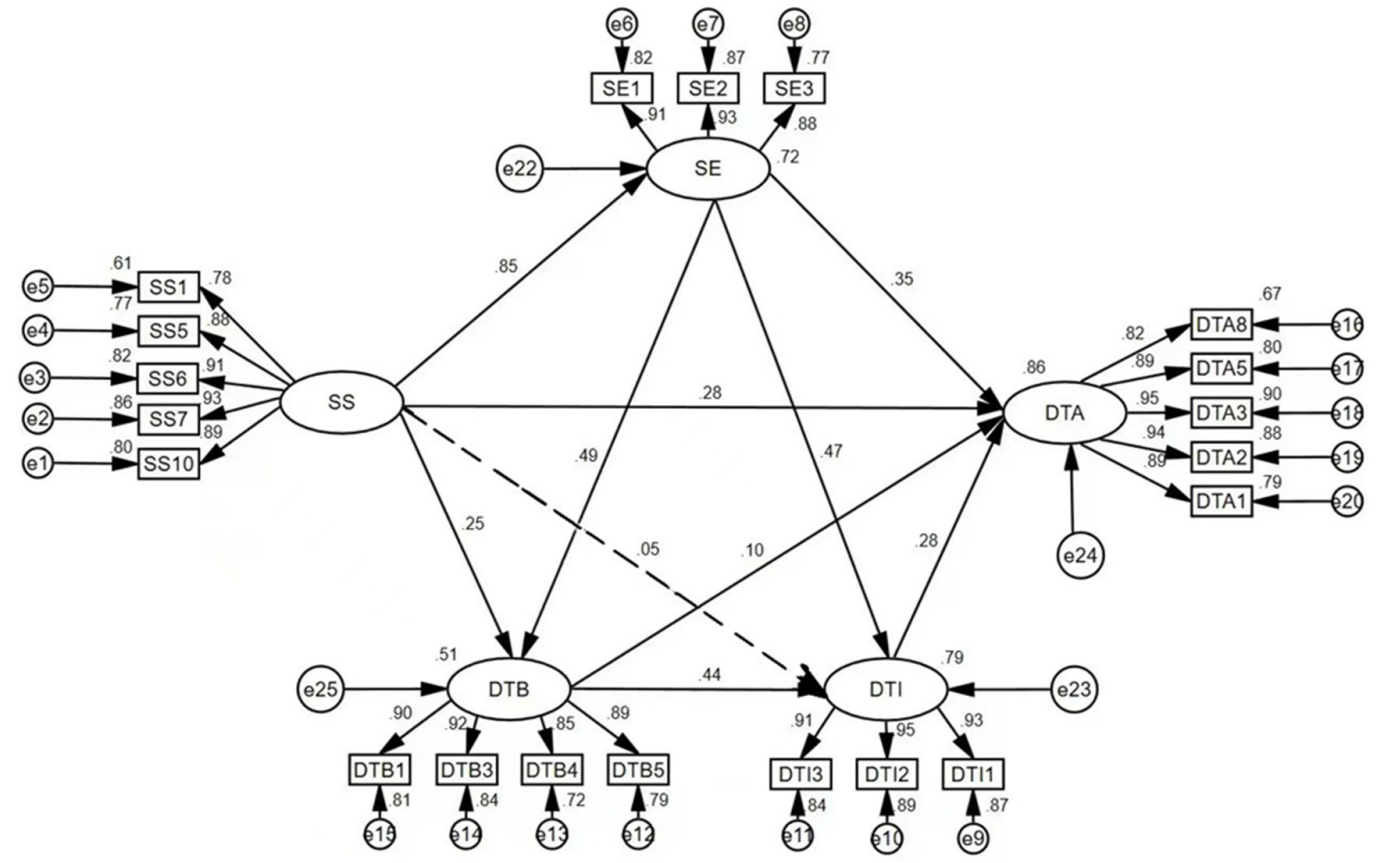
Results of the hypothesis testing for the path relationships in the structural equation model.

The results of the path analysis are shown in Table 3 and Figure 2. School support was significantly and positively associated with PE teachers' digital teaching adaptation (β = 0.276, *p* < 0.001), thereby supporting H1. It also significantly predicted PE teachers' self-efficacy (β = 0.847, *p* < 0.001) and digital teaching beliefs (β = 0.252, *p* < 0.01), confirming H2 and H4. However, the direct path from school support to digital teaching intention was not statistically significant (β = 0.055, *p* >0.05), thus H7 was not supported. Self-efficacy significantly predicted both digital teaching adaptation (β = 0.346, *p* < 0.001) and digital teaching beliefs (β = 0.487, *p* < 0.001), as well as digital teaching intention (β = 0.475, *p* < 0.001), supporting H3, H5, and H8. Furthermore, digital teaching beliefs were found to significantly predict both digital teaching adaptation (β = 0.101, *p* < 0.05) and digital teaching intention (β = 0.438, *p* < 0.001), confirming H6 and H9. Finally, digital teaching intention significantly and positively predicted digital teaching adaptation (β = 0.281, *p* < 0.001), providing support for H10.

**Table 3:** Path test results of each factor.

**Hypothesis**	**Estimate**	**S.E**.	**C.R**.	***P***	**Result**
H1:SS → DTA	0.276	0.040	5.530	^***^	Supported
H2:SS → SE	0.847	0.037	20.781	^***^	Supported
H3:SE → DTA	0.346	0.055	5.605	^***^	Supported
H4:SS → DTB	0.252	0.066	3.055	^**^	Supported
H5:SE → DTB	0.487	0.075	5.790	^***^	Supported
H6:DTB → DTA	0.101	0.043	2.381	^*^	Supported
H7:SS → DTI	0.055	0.048	0.928	0.354	Not Supported
H8:SE → DTI	0.475	0.058	7.377	^***^	Supported
H9:DTB → DTI	0.438	0.044	10.196	^***^	Supported
H10:DTI → DTA	0.281	0.057	4.950	^***^	Supported

^***^p < 0.001; ^**^p < 0.01; ^*^ p < 0.05.

### 4.5 Mediation analysis

A bootstrap test was conducted to examine the mediating effects of self-efficacy, digital teaching intention, and digital teaching beliefs. A total of 2,000 resamples were drawn to calculate direct, indirect, and total effects. As shown in Table 4, the confidence interval (CI) for the direct path of school support on PE teachers' digital teaching adaptation was [0.121, 0.426], with *p* < 0.001, indicating a significant direct effect. The CI for the indirect path from school support to self-efficacy to PE teachers' digital teaching adaptation was [0.130, 0.479], *p* < 0.001, confirming the significant mediating role of self-efficacy. The indirect effect through school support to digital teaching beliefs to PE teachers' digital teaching adaptation had a CI of [0.000, 0.079], *p* > 0.05, indicating a non-significant mediation via digital teaching beliefs. Similarly, the path from school support to digital teaching intention to PE teachers' digital teaching adaptation yielded a CI of [−0.020, 0.072], *p* > 0.05, suggesting that digital teaching intention did not serve as a significant mediator. However, the chain mediation via school support to self-efficacy to digital teaching intention to PE teachers digital teaching adaptation was significant, with a CI of [0.034, 0.255], *p* < 0.01. The chain path through school support to self-efficacy to digital teaching beliefs to PE teachers' digital teaching adaptation also showed significance (CI = [−0.004, 0.129], *p* < 0.01). In addition, the chain mediation through school support to digital teaching beliefs to digital teaching intention to PE teachers' digital teaching adaptation was significant (CI = [0.004, 0.093], *p* < 0.01). Lastly, the extended chain mediation path through school support to self-efficacy to digital teaching beliefs to digital teaching intention to PE teachers' digital teaching adaptation was also significant, with a CI of [0.020, 0.122], *p* < 0.001.

**Table 4:** Bootstrap analysis of the mediation effect.

**Path**	**SE**	**Estimate**	Bootstrap				***P***
			Percentile 95% CI		BC 95% CI		
			**Lower**	**Upper**	**Lower**	**Upper**	
DE							
SS → DTA	0.077	0.276	0.110	0.421	0.121	0.426	0.001
IE							
SS → SE → DTA	0.089	0.293	0.118	0.474	0.130	0.479	0.001
SS → DTB → DTA	0.019	0.026	−0.009	0.065	0.000	0.079	0.054
SS → DTI → DTA	0.022	0.015	−0.029	0.064	−0.020	0.072	0.308
SS → SE → DTI → DTA	0.056	0.113	0.034	0.252	0.034	0.255	0.003
SS → SE → DTB → DTA	0.033	0.042	−0.011	0.116	−0.004	0.129	0.070
SS → DTB → DTI → DTA	0.021	0.031	0.001	0.084	0.004	0.093	0.023
SS → SE → DTB → DTI → DTA	0.026	0.051	0.016	0.114	0.020	0.122	0.001
TE							
SS → DTA	0.023	0.847	0.799	0.889	0.796	0.886	0.001

## 5 Discussion

This study aimed to investigate the relationship between school support and digital teaching adaptation among PE teachers, with a particular focus on the mediating roles of self-efficacy, digital teaching intention, and digital teaching beliefs.

First, the results of this study demonstrate that school support was significantly, directly and positively associated with on PE teachers' digital teaching adaptation. This indicates that the school-level support system plays a crucial role in facilitating teachers' engagement in the digital transformation of education. This finding is consistent with previous research. Scherer et al. (2019) conducted a meta-analytic structural equation modeling study showing that infrastructure and training resources within school environments significantly predict teachers' technology adoption behaviors. Similarly, Claro et al. (2024) emphasized in their systematic review that resource investment and professional development at the school level are essential external conditions for fostering teachers' digital teaching innovation. Furthermore, school support was found to be significantly associated with teachers' self-efficacy and digital teaching beliefs, whereas its direct association with digital teaching intention was not supported. This result suggests that although school support can provide teachers with necessary resources, institutional guarantees, and technological conditions, such external support does not automatically translate into teachers' instructional intentions. This is inconsistent with some previous research and our assumptions. Previous research has shown that school support are related to the continuance intention to teach online for teachers (Chou and Chou, 2021), and when schools provide adequate technical resources and necessary training, teachers' intention to engage in digital teaching can be significantly improved (Saiz-González et al., 2025). However, some previous theory and research have also presented findings that are consistent with this study. For instance, Bandura's social cognitive theory similarly supports this view, suggesting that school support, as an environmental factor, does not directly drive behavioral intentions; rather, it must be internalized through individuals' psychological and cognitive processes in order to facilitate the formation of behavioral intentions (Bandura, 1986). Previous research has found that pre-service teachers' behavioral intention to use technology was indirectly predicted by facilitating conditions and mediated by attitude toward usage (Teo, 2010). Overall, the present study confirms that school support functions as a key external variable within the ecosystem of educational digitalization. It not only provides the necessary material and institutional foundation but also establishes an enabling environment that nurtures teachers' ongoing professional growth and adaptive engagement in digital teaching.

Secondly, self-efficacy partially mediates the association between school support and PE teachers' digital teaching adaptation. As established earlier, school support is significantly and positively associated with teachers' digital teaching adaptation. Further analysis revealed that school support significantly predicts teachers' self-efficacy, which in turn facilitates their digital teaching adaptation. This finding indicates that self-efficacy functions as an intermediary mechanism linking school support and teachers' adaptive behavior, which is consistent with social cognitive theory. Existing empirical studies further substantiate this mechanism. For instance, Thomas (2023) found that systematic school-based training in sports data analysis significantly strengthened PE teachers' self-efficacy, thereby enhancing their adaptability in digital instruction. Similarly, Ye et al. (2022) provided empirical evidence that organizational support enhances teachers' self-efficacy, which in turn promotes their integration of technology into instruction. These findings suggest that in promoting the digital transformation of physical education, schools should shift their focus from “providing” to “empowering.” Beyond the mere provision of resources and platforms, effective school support should prioritize the cultivation of teachers' self-efficacy through high-quality professional development, cognitive support, and motivational stimulation, facilitating the internalization and sustained transformation of digital teaching behaviors. By contrast, although school support significantly predicted PE teachers' digital teaching beliefs, and digital teaching beliefs also significantly predicted their digital teaching adaptation, the bootstrap test did not provide support for a significant mediator effect of digital teaching beliefs between school support and teachers' digital teaching adaptation. This finding suggests that digital teaching beliefs may primarily reflect teachers' evaluations of the value and feasibility of digital instruction, but such evaluations alone may be insufficient to sustain a stable transmission from external support to adaptive teaching behavior. Prior studies have similarly emphasized that teachers' beliefs represent a complex psychological construct, and that their translation into instructional practice is often contingent upon contextual factors such as school culture and resource availability (Bice and Tang, 2022). Furthermore, although digital teaching intention was significantly associated with digital teaching adaptation, the direct association between school support and digital teaching intention was not significant, thereby preventing the establishment of a significant indirect effect.

Finally, the results indicate that school support is significantly associated with PE teachers' digital teaching adaptation through multiple serial mediation pathways. One path indicates that teachers' self-efficacy and digital teaching intention play a chain mediation role between school support and digital teaching adaptation. As discussed earlier, the direct path from school support to digital teaching intention is not statistically significant, and the mediation role of digital teaching intention between school support and digital teaching adaptation is not supported. However, school support is significantly associated with higher levels of teachers' self-efficacy, which was subsequently associated with their digital teaching intention, thereby linking school support to adaptive instructional behavior. This finding suggests that school support is more likely to be first internalized as teachers' confidence in their capabilities, which subsequently facilitates the formation of clearer digital teaching intentions and, in turn, relates to adaptive teaching behavior. This further suggests the significance of teachers' self-efficacy in the transformation of external support into teachers' behavior. Meanwhile, it explains why school support has no significant direct effect on digital teaching intention in this study, indicating that the influence of school support on digital teaching intention requires certain internal psychological variables such as self-efficacy, rather than through a direct pathway. Prior research has similarly highlighted teachers' self-efficacy as drivers of their intention to use technology during emergency remote teaching (Georgiou et al., 2023). Another path shows that digital teaching beliefs and digital teaching intention also form a chain mediation pathway between school support and digital teaching adaptation. Although the mediating roles of both digital teaching beliefs and digital teaching intention in the relationship between school support and digital teaching adaptation are not supported, school support is indirectly associated with teachers' digital teaching adaptation through the combined effect of digital teaching beliefs and digital teaching intention. Prior research similarly indicates that supportive school environments can shape teachers' digital teaching beliefs and, through their influence on teaching intention, further facilitate teachers' adaptation and adjustment in digital instruction (Scherer et al., 2019). In addition, the present findings reveal that school support is indirectly associated with PE teachers' digital teaching adaptation through the sequential path of self-efficacy, digital teaching beliefs, and digital teaching intentions, highlighting the cumulative influence of internal psychological mechanisms. In summary, the influence of external school support does not appear to be linear; rather, it operates through internalization into teachers' self-efficacy, beliefs and intentions, which are in turn associated with sustained adaptive teaching behavior. This finding emphasizes that it's necessary to adopt a systematic approach to promote the synergistic effect of external school support and internal psychological factors of teachers to enhance PE teachers' digital teaching adaptation. Teachers' self-efficacy, digital teaching beliefs, and digital teaching intention and digital teaching intention are all crucial internal psychological factors in the path of school support influencing teachers' digital teaching adaptation, among which digital teaching intention is an essential psychological factor for external support to transform into teachers' digital teaching adaptation. Meanwhile, in the path of school support influencing teachers' adaptation to digital teaching, there is a progressive mechanism of internal psychological factors among teachers, following a continuous psychological chain from capability confidence to value recognition and then to behavioral intention, which is consistent with the core propositions of social cognitive theory.

## 6 Conclusion and limitations

The study reveals that school support is significantly and directly associated with digital teaching adaptation among PE teachers. Self-efficacy serves as a partial mediator in the relationship between school support and digital teaching adaptation. Self-efficacy, digital teaching beliefs, and digital teaching intention jointly constitute a serial mediation mechanism linking school support to digital teaching adaptation among PE teachers.

This study creatively applies SCT to the context of PE teachers' digital teaching adaptation, extending its theoretical scope and offering a new analytical perspective. Considering the digital context, and building upon the core logic of SCT, the study incorporates the “intention” variable from the Technology Acceptance Model (TAM), which is directly associated with behavior into the “personal factors” dimension. This results in a reconstructed “personal factors” construct encompassing self-efficacy, digital teaching beliefs, and digital teaching intention, while examining the interrelationships among these variables. This enriches the composition of personal factors within SCT and enhances its explanatory power in technology-mediated and digital contexts. It also provides strong empirical evidence for the critical role of school support in promoting digital transformation, with self-efficacy and digital teaching intention identified as key mediators to guide educational policy and practice.

Despite offering valuable insights, this study has limitations that warrant cautious interpretation and suggest directions for future research. First, the cross-sectional design limits the ability to make causal interpretations. Longitudinal studies examining changes in school support and teachers' psychological factors over time would provide clearer evidence of the dynamic mechanisms underlying digital teaching adaptation. Second, although the school support covered five different dimensions when conducting the measurement in the present study, taking into account the focus of the research question, model complexity, and the number of measurement items, it was operationalized as a single higher-order construct in data analysis. Future research may further distinguish its specific components (e.g., technical support, infrastructure, curriculum support, and school climate) to compare their potentially differentiated effects on teachers' digital teaching adaptation. Third, although procedural remedies were implemented and the common method variance test was conducted, it still has certain limitations. The study relies primarily on quantitative, self-reported data from physical education teachers, which may raise concerns regarding common method variance. Specifically, when all variables are collected from the same respondents at the same time point using the same measurement instrument, respondents' perception of the questionnaire's logical structure, acquiescence tendencies, and social desirability effects may artificially inflate inter-variable correlations, leading to overestimated path coefficients and potentially obscuring the true nature of the relationships under investigation. Although the test results indicate that common method variance is not a serious concern in the present study, future research could further employ multiple methods, such as multi-source data collection and longitudinal multi-wave measurement designs to more effectively mitigate the potential influence of common method variance, thereby enhancing the external validity and robustness of the findings. Additionally, the study was conducted in a single cultural context, limiting the generalizability of its findings. Cross-cultural comparisons are needed to assess whether the identified mechanisms are universally applicable or context-specific, thereby enhancing the global relevance of the proposed model.

## Data Availability

The raw data supporting the conclusions of this article will be made available by the authors, without undue reservation.
