# Supplementary material for: Relationship between school support and digital teaching adaptation among physical education teachers: the mediating roles of self-efficacy, digital teaching beliefs and teaching intention
Source: Front Psychol. 2026 Mar 18;17:1786873. doi: 10.3389/fpsyg.2026.1786873 (PMC13038524; doi:10.3389/fpsyg.2026.1786873)
Supplement: Supplementary file 1 [file Supplementary_file_1.docx]

**Statistical Procedures for Item Removal**

Phase 1 item removal was conducted during CFA based on substantial correlated residuals and modification indices. Phase 2 refinement was conducted after embedding the measurement model into the full SEM to address persistent residual dependencies.

**Phase 1 item removal**

Item removal in Phase 1 was conducted sequentially based on factor loadings, high correlated residuals and modification indices identified in the CFA. While standardized factor loadings below 0.70 were established as a removal criterion, all items in the initial model exhibited strong loadings, so no items were removed for this reason. Instead, removal was based on modification indices greater than 10 (p<0.001) indicating high residual correlations, which violated the assumption of local independence and potentially compromised construct validity. Items were removed with theoretical considerations prioritized at each step to ensure alignment with the conceptual definition of the latent construct.

**School Support**

Table A1 Comparison of CFA Model Fit Before and After Item Removal

| Model | χ²/df | CFI | GFI | TLI | RMSEA | SRMR | No. of Items |
| --- | --- | --- | --- | --- | --- | --- | --- |
| Initial CFA model | 10.821 | 0.925 | 0.843 | 0.903 | 0.155 | 0.0365 | 10 |
| Revised CFA model 1 | 8.225 | 0.950 | 0.890 | 0.950 | 0.133 | 0.0315 | 9 |
| Revised CFA model 2 | 4.453 | 0.980 | 0.941 | 0.971 | 0.092 | 0.0204 | 8 |
| CFA after Phase 1 removal | 2.934 | 0.990 | 0.973 | 0.986 | 0.069 | 0.0156 | 7 |

Table A2 Statistical Characteristics of Removed Items

| Construct | Item Code | Std. Loading | Error Variance | High MI (with) | Reason for Removal |
| --- | --- | --- | --- | --- | --- |
| SS | SS9 | 0.864 | 0.75 | 98.672 (SS8) 29.842 (SS10)  16.770 (SS5) | High residual correlation |
| SS | SS2 | 0.823 | 0.66 | 94.908 (SS1)  25.415 (SS7) | High residual correlation |
| SS | SS3 | 0.867 | 0.75 | 21.988 (SS4) | High residual correlation |

**Digital Teaching Belief**

Table A3 Comparison of CFA Results Before and After Item Removal

| Model | χ²/df | CFI | GFI | TLI | RMSEA | SRMR | No. of Items |
| --- | --- | --- | --- | --- | --- | --- | --- |
| Initial CFA model | 7.509 | 0.976 | 0.948 | 0.961 | 0.126 | 0.0211 | 6 |
| Revised CFA model | 5.208 | 0.989 | 0.976 | 0.979 | 0.102 | 0.0136 | 5 |
| CFA after Phase 1 removal | 0.020 | 1.000 | 1.000 | 1.000 | 0.000 | 0.0007 | 4 |

***Note.*** *The “CFA after Phase 1 removal” model represents a highly parsimonious solution, retaining only 4 items with the strongest theoretical and empirical fit. The near-perfect fit indices (*χ²*(2)=0.041,p=0.980，CFI = 1.000，GFI = 1.000，AGFI = 1.000，RMSEA = 0.000) reflect the model’s extreme simplicity (df=2).* *When combined with factor loadings and reliability, the scale demonstrates excellent internal consistency and convergent validity, with items strongly representing the latent construct.*

Table A4 Statistical Characteristics of Removed Items

| Construct | Item Code | Std. Loading | Error Variance | High MI (with) | Reason for Removal |
| --- | --- | --- | --- | --- | --- |
| DTB | DTB2 | 0.856 | 0.73 | 18.547 (DTB1)  13.963 (DTB4)  11.787 (DTB6) | High residual correlation |
| DTB | DTB6 | 0.877 | 0.77 | 10.938 (DTB5) | High residual correlation |

**Digital Teaching Adaptation**

Table A5 CFA Model Fit Before and After Item Removal

| Model | χ²/df | CFI | GFI | TLI | RMSEA | SRMR | No. of Items |
| --- | --- | --- | --- | --- | --- | --- | --- |
| Initial CFA model | 4.985 | 0.980 | 0.939 | 0.972 | 0.099 | 0.0186 | 8 |
| Revised CFA model 1 | 4.180 | 0.987 | 0.957 | 0.981 | 0.088 | 0.0141 | 7 |
| CFA after Phase 1 removal | 3.222 | 0.993 | 0.977 | 0.988 | 0.074 | 0.0136 | 6 |

Table A6 Statistical Characteristics of Removed Items

| Construct | Item Code | Std. Loading | Error Variance | High MI (with) | Reason for Removal |
| --- | --- | --- | --- | --- | --- |
| DTA | DTA7 | 0.828 | 0.69 | 22.917 (DTA8)  17.578 (DTA2) | High residual correlation |
| DTA | DTA4 | 0.937 | 0.88 | 18.926 (DTA6) | High residual correlation |

**Phase 2 refinement**

After embedding the refined measurement model into the full SEM, persistent residual dependencies were observed between select items. These dependencies were addressed through additional item removal, as correlating residual errors without theoretical justification was deemed methodologically unsound. Removal decisions were guided by both high modification indices (MI > 10) and theoretical relevance, ensuring the final SEM maintained both statistical fit and conceptual coherence.

Table B1 SEM-Embedded Measurement Refinement

| Model | χ²/df | CFI | GFI | TLI | RMSEA | SRMR |
| --- | --- | --- | --- | --- | --- | --- |
| SEM with CFA-refined items | 2.785 | 0.966 | 0.884 | 0.961 | 0.066 | 0.031 |
| Final SEM model | 2.432 | 0.977 | 0. 913 | 0.973 | 0.059 | 0.026 |

Table B2 Items Removed in SEM Stage

| Construct | Item | Std. Loading | MI (with item) | Reason |
| --- | --- | --- | --- | --- |
| SS | SS8 | 0.824 | 18.338 (DTA2) | Residual dependency |
| DTA | DTA6 | 0.861 | 17.637 (DTB4) | Residual dependency |
| SS | SS4 | 0.840 | 15.426 (SS5) | Residual dependency |

***Note.*** *Items were removed by both high modification indices (MI>10) and theoretical relevance to ensure the final model maintained conceptual coherence.*

Table B3 Final Reliability and Validity of Constructs

| Construct | Cronbach’s α | CR | AVE |
| --- | --- | --- | --- |
| School Support | 0.943 | 0.944 | 0.772 |
| Digital Teaching Belief | 0.937 | 0.937 | 0.789 |
| Digital Teaching Adaptation | 0.954 | 0.955 | 0.810 |
